# Primary caregivers’ experiences of caring for people living with dementia in Ghana: a phenomenological study

**DOI:** 10.1186/s12877-024-04894-6

**Published:** 2024-04-01

**Authors:** Precious Adade Duodu, Bibha Simkhada, Joshua Okyere, Ransford Akrong, Caroline Barker, Warren Gillibrand, Padam Simkhada

**Affiliations:** 1 Department of Nursing, School of Human and Health Sciences, University of Huddersfield, Queensgate, Huddersfield, UK; 2 Department of Population and Health, University of Cape Coast, Cape Coast, Ghana; 3 School of Nursing and Midwifery, College of Health Sciences, Kwame Nkrumah University of Science and Technology, Kumasi, Ghana; 4 Educational Assessment and Research Center, Osu, Accra Ghana; 5 School of Human and Health Sciences, University of Huddersfield, Queensgate, Huddersfield, UK

**Keywords:** Caregivers, Dementia, Alzheimer’s, Qualitative research

## Abstract

**Background:**

Dementia is often associated with functional impairments that limit the independence of persons living with dementia (PwD). As such, many PwD often require a higher level of support provided by persons referred to as caregivers. Such caregiving activities tend to strain and stress the caregiver. Nonetheless, Ghana lacks empirical evidence and understanding of the effects of caring for PwD on the lives of primary caregivers. To help narrow this knowledge gap, we explored the perspectives of primary caregivers about the impacts of caring for PwD in Ghana.

**Methods:**

Using a descriptive phenomenological design, we conducted in-depth interviews with primary caregivers in the Ashanti region, Ghana. A semi-structured interview guide was used as the data collection instrument. The data analysis followed Collazi’s thematic analysis framework. All coding and categorization were done in NVivo-12.

**Results:**

Five themes emerged from the analysis. These themes included (a) sacrifice of personal interests, and time commitments; (b) financial strain and negative impact on job; (c) feelings of stress and burnout; (d) experience of abuse and stigma; and (e) perceived blessing of caregiving.

**Conclusion:**

The study’s findings resonate with existing literature, highlighting the consistent struggles faced by caregivers. Sacrificing personal interests, navigating financial strains, and grappling with stress and burnout emerged as pervasive themes. We conclude that despite the negative impacts of caring for PwD, caregivers perceived their role as associated with blessings, deriving positive meaning and fulfilment from their caregiving journey. This study underscores a need to build more compassionate communities in rural settings of Ghana.

**Supplementary Information:**

The online version contains supplementary material available at 10.1186/s12877-024-04894-6.

## Background

The dynamics of world’s population is rapidly changing with many people living longer than before [1]. Amidst this improvement in life expectancy and longevity comes with chronic and degenerative morbidities. Dementia is one of the degenerative conditions that often accompanies an ageing population [2]. According to the World Health Organization (WHO) [3], there are over 55 million people living with dementia (PwD) across the globe. Of this number, nearly 60% are reported in low-and-middle-income countries (LMICs) [3]. In sub-Saharan Africa (SSA), the prevalence of dementia is projected to surpass 7.6 million cases by the end of 2050 [4]. This makes dementia a growing threat to the healthcare system of SSA. Although Ghana lacks comprehensive data on dementia, it is estimated that the condition is prevalent among 5% of the overall population [5]. Consequently, there have been an increasing concern for dementia and its nuances in Ghana.

Literature [3, 6, 7] shows that dementia is often associated with functional impairments that limits the independence of the PwD. As such, many PwD often require a higher level of support which is provided by persons referred to as caregivers. Caregiver refers to a person who assists or guides a person in need with the necessary physical and psychological care [8]. In this study, a caregiver is anyone who helps with the physical and psychosocial care of PwD on a consistent basis, and is recognized by the PwD and the family for that role. Usually, caregivers for PwD are family member, spouse or children; however, there are times that the individual may be a health worker or social worker who is paid to render caregiving services [8].

It is important to acknowledge that without caregivers, PwD are more likely to have poorer quality of life [6, 7]. This is because caregivers support the PwD to seek healthcare, adhere to treatment regimen, and have a healthy dietary behaviour. For instance, Brodaty and Donkin [6] assert that caregivers *“provide hands-on care, dressing, assisting with finances and other daily activities”* of PwD. Such caregiving activities tends to strain and stress the caregiver. Nonetheless, Ghana lacks empirical evidence and understanding of the effects of caring for PwD on the life of primary caregivers. The extant literature in Ghana has mainly focused on understanding dementia research needs and gaps [5], the caregiver’s needs [9], utilization of technology to improve the quality of life of PwD [10], and the lived experiences of PwD [11, 12]. To the best of our knowledge, and after extensive literature search, there is currently no published empirical evidence that qualitatively explores the lived experiences of primary caregivers of PwD in relation to the impact of assuming caregiving role in Ghana. Hence, the following questions remain unanswered: (a) What are the impacts of caring for PwD in Ghana? (b) Does caregiving for PwD produce both positive and negative impacts? Addressing these questions is critical to gaining valuable and deeper insights into the nuances of caregiving for PwD in a setting like Ghana where dementia is shrouded in myths, misperceptions, and spiritualism [11]. To help narrow this knowledge gap, we explored the perspectives of primary caregivers about the impact of caring for PwD in Ghana.

## Methods

### Study design

In line with the objective of the study, we adopted a qualitative research methodology. Particularly, a descriptive phenomenological approach was selected. This research design was chosen due to its focus on investigating and portraying the genuine lived experiences of individuals, as opposed to relying solely on perceived interpretations of a specific phenomenon [13]. Descriptive phenomenology was deemed the most appropriate design for our study since the focus was on unearthing the lived experiences of caregivers in terms of the impact that caring for PwD.

### Ethical considerations

Ethical approval was obtained from the Ghana Health Service Ethics Review Committee (GHS-ERC) [ID Number: GHS-ERC: 005/02/23] and the School Research Ethics and Integrity Committee (SREIC), University of Huddersfield, United Kingdom (SREIC Reference: SREIC_ExtApp_2023_001). Also, approvals were obtained from the respective district/municipal/metropolitan health directorates where the sampled healthcare facilities were situated. Furthermore, we obtained permission from all the healthcare facilities where the study participants were recruited. Additionally, oral and written informed consent was obtained from each participant. The rights and responsibilities as a participant were read by, or read and explained to (where necessary), each primary caregiver to inform them to decide whether to participate in the study. Participants were assured of confidentiality, data protection, anonymity, and the right to withdraw from the study without any consequence, loss of benefit, care or treatment to them or their relatives with dementia in the facilities where they were recruited. The study was performed in accordance with the ethical standards as laid down in the 1964 Declaration of Helsinki and its later amendments or comparable ethical standards.

### Setting

The study was designed as a multicentre study where healthcare facilities that provided healthcare to PwD in some selected districts in the Ashanti region were selected. This is a multicentre study involving eight health facilities namely Ejisu Government Hospital, Juaben Government Hospital, University Hospital– KNUST, Kumasi South Hospital, Manhyia District Hospital, Onwe Government Hospital, and Tafo Government Hospital.

### Sample and recruitment

Purposive sampling technique was adopted to sample and recruit primary caregivers. The rationale behind the use of purposive sampling was to deliberately select participants who possess a wealth of first-hand experience in providing care for PwD in Ghana. Given the nuanced nature of caregiving and the specific focus on exploring the impact from the perspective of primary caregivers, purposive sampling allowed for a targeted selection process. This approach ensured that participants had direct involvement and deep insight into the caregiving phenomenon, thereby enriching the depth and authenticity of the data collected [14, 15]. Participants were deemed eligible to participate in the study if the met the following inclusion criteria: (a) be an unpaid primary caregiver of a PwD (i.e., a family member, such as a spouse, adult child, or other close relative, who takes on the role of coordinating and managing the daily care needs of the PwD on a consistent and ongoing basis); (b) have face-to-face contact with this person with dementia for a minimum of once a week; (c) be an adult, that is, aged 18 years and above. Any individual who did not fulfil the complete criteria was excluded from the study.

### Data collection

A semi-structured interview guide was used as the data collection tool (see Supplementary File 1). This guide was informed by prior literature on caregiving for PwD [16–20]; guiding how major questions and probing questions are framed. All the individual, in-depth interviews were conducted in-person at the various healthcare facilities. First, data collectors who were trained nurses who had completed their bachelor’s degree were recruited and trained. These nurses were all indigenes of the Ashanti region and were proficient in speaking, writing and understanding Twi (the dominant local language of the Ashanti Region). There were two weeks intensive training which covered issues relating to the data collection tool, purpose and objectives of the study, ethics and how to screen and select unpaid primary caregivers as participants. After the training, the data collectors were assigned to the respective healthcare facilities. The data collection period was 17th April to 31st May 2023. The first week was used to identify and screen prospective participants. Unpaid primary caregivers identified in the first week of the data collection were provided with information and an oral explanation of what the study entailed, the procedures, benefits, and their rights/responsibilities should they choose to participate. These unpaid primary caregivers were followed up in the subsequent week for their response. All thirty (30) unpaid primary caregivers that were screened agreed to participate in the study. The data collectors then scheduled a time with the caregivers to have the interviews. All participants agreed that the healthcare facility where they were recruited be used as a venue for the interviews. The interviews were conducted in English and Twi based on the preference of the participants. Specifically, 21 of the interviews were in English while the remaining 9 in Twi. None of the participants withdrew from the study. The duration of each interview averaged forty-five (45) minutes. Each interview was was audio-recorded by the data collectors.

### Data analysis

Data analysis was guided by Colaizzi’s thematic analysis framework [21, 22]. The audio data were transcribed verbatim. Interviews conducted in Twi were translated by the particular individual who collected the data. To validate the quality of the translated transcripts, we hired the services of a professional Ghanaian language translator to compare the translated transcript with the audio data. Some editing was made to improve on the translated transcripts before the formal analysis. The final translated transcripts were validated by PAD, JO, and RA. JO carried out the initial formal analysis supported by PAD and RA; first the transcripts were read several times as a way of getting familiarized with the data. The transcripts were then imported into QSR NVivo-12 for data management and coding. Key phrases, statements, and expressions relevant to the caregiving experience were identified within the transcripts and assigned initial codes [21, 22]. The significant statements were coded to create condensed meaning units, capturing the essence of participants’ descriptions and emotions. Assigned codes were reviewed and grouped into themes that reflected common aspects of the caregiving experience. Subsequently, the authors discussed and elaborated upon the themes to create clear and comprehensive descriptions that communicated the depth and breadth of participants’ experiences. This validation process continued until a consensus was agreed regarding the final themes. The findings were reported using selected extracts from the transcripts to exemplify and substantiate the identified themes [22].

### Rigor and trustworthiness

In accordance with Guba and Lincoln’s propositions [23] that underscore the critical importance of rigor in qualitative research, the present study meticulously incorporated measures to uphold the credibility, confirmability, transferability, and dependability of our findings. To ensure credibility, we repeated readings and in-depth immersion in participants’ narratives [24]. Additionally, only verbatim quotes were used to exemplify the perspectives of the participants. We ensured transferability by providing a detailed description of our methodology and study area [25]. Therefore, other researchers who seek to work in similar contexts can rely on our methodology for guidance. Throughout the data collection process, we held debriefing sessions with the research assistants. This helped to clarify any inconsistencies and ensure that the interviewers did not introduce their biases into the research process, hence, helping to achieve confirmability and dependability. Also, we kept track of the audio data and transcripts for confirmability purposes.

## Findings

### Socio-demographic characteristics

Of the thirty (30) primary caregivers who participated in the study, 8 (26.7%) were males while 22 (73.3%) were females. Also, 9 (30%) were between the ages of 25 and 34 years, 6 (20%) were aged 35–44 years, and half (50%) of them were 45 years or older. Regarding their highest educational level, 2 (6.7%), 6 (20.0%), 6 (20.0%), and 16 (53.3%) had none, Junior High School, Senior High School, and Tertiary qualifications. About 12 (40.0%) were single, 5 (16.7%) were married, and 13 (43.3%) were divorced. The study recognized daughters (53.3%) and sons (23.3%) of PwD as the predominant primary caregivers, respectively (see Table 1).

**Table 1:** Sociodemographic characteristics of study participants

Characteristics	Sample size (*N* = 30)	Percentages
***Sex*** Male female	8 22	26.7% 73.3%
***Age (in years)*** *2*5–34 35–44 ≥ 45	9 6 15	30.0% 20.0% 50.0%
***Highest educational level*** None Junior High School Senior High School Tertiary	2 6 6 16	6.7% 20.0% 20.0% 53.3%
***Marital status*** Single Married Divorced	12 5 13	40.0% 16.7% 43.3%
***Relationship with PwD*** Daughter Son Daughter in-law Niece Wife Sister House help	16 7 2 1 2 1 1	53.3% 23.3% 6.7% 3.3% 6.7% 3.3% 3.3%

### Main findings

Five themes emerged from the analysis. These themes included (a) sacrifice of personal interests, and time commitments; (b) financial strain and negative impact on job; (c) feelings of stress and burnout; (d) experience of physical/verbal abuse and stigma; and (e) perceived blessing of caregiving.

### Sacrifice of personal interests, and time commitments

A recurring theme in this study was that primary caregivers had to sacrifice their personal interests. The participant revealed that their social engagements had diminished as a consequence of caring for their mother with dementia. This reduction in social activities was attributed to the time and energy dedicated to caregiving duties.

*“So, if I will go somewhere I would need someone to come here and take care of her for me else I don’t get any chance of going anywhere, and if you try sending her to somewhere and instruct her to wait for you, by the time you are back you wouldn’t find her there. So am unable to go anywhere because of her situation”* (Daughter of PwD attending Juaben government hospital, 35–44 years).

*“Even when I am ready to go to church on Sundays and he asks me to return and do something for him, I do not go to church and rather pay attention to him else he gets worried”* (Son of PwD attending Kumasi south government hospital, 45–49 years).

Furthermore, the participants divulged experiencing a sense of preoccupation during social gatherings. The fear of potential issues arising at home compelled them to ensure that domestic chores were completed before attending any event. Failing to attend to these responsibilities before leaving for social occasions led to lingering concerns about the wellbeing of the care recipient. The quote below reflects this finding:

*“I will say that my social life has reduced due to my mother and about my work too, I am able to come to work early, other times, I come late because I have to take care of stuff at home first…I sometimes want to take care of all the chores I need to perform at home before I leave for these occasions else even as I am at these gatherings, my mind would still be at home worried about all the things that could possibly go wrong”* (Daughter of PwD attending Tafo government hospital, 25–34 years).

### Financial strain and negative impact on job

The analysis revealed that primary caregivers of PwDs faced financial strains as a result of assuming caregiving responsibilities. Primarily, this financial constrain emanated from two reasons: cost of healthcare and interruption of work. The participants asserted that caring for their PwD was financially challenging as they had to bear direct cost of healthcare such as the cost of medications, as well as ancillary health expenditure like transportation cost. Beyond the cost of transportation and medications, the participants revealed that assuming caregiving role for PwD limited their capacity to work. Some caregivers expressed that their care recipient’s inability to communicate basic needs, such as thirst or other desires, significantly hindered their capacity to continue working. This communication barrier necessitated their continuous presence and attention to the care recipient’s needs, rendering traditional work arrangements unfeasible. The participants further articulated that whenever these caregiving crises occurred, they were compelled to halt their work commitments to prioritize the care recipient’s well-being. This reactive nature of caregiving, necessitated by unpredictable health events, resulted in regular interruptions to their work routine. Consequently, the participant’s employment consistency was compromised

*“It was not easy at all for us when her illness started, because you need a lot of money too for her drugs. I am a graphic designer by profession, I lost two contracts because I had to leave her to Accra to execute it but I could not go”* (Son of PwD attending Kumasi south government hospital, 45–49 years ).

*“I am unable to go to work because she cannot even tell me when she is thirsty, or want to do anything else. My finances diminished when I started taking care of her. Costs of transportation is draining. The distance from the bus stop is quite far so we are not able to walk we need to take a taxi. We are really struggling with finances”* (Wife of PwD attending Kumasi south government hospital, 60 years).

Another participant expressed that:

*“I have not been able to go to work this week, he has not been well, so I have to stay at home and take care of him. Whenever his crisis comes, I have to stop work and come and take care of him, and that is why my finances are not good”* (Son of PwD attending Tafo government hospital, 30–34 years).

### Feelings of stress and burnout

In the view of the participants, the dual role as a caregiver and a worker led to notable fatigue and feelings of being overwhelmed. Primary caregivers of PwD expressed feelings of burden and worry, particularly when anticipating upcoming hospital visits. As a result of the feelings of stress and burnout, the participants divulged experiencing discouragement and moments of contemplation about relinquishing their caregiving responsibilities. Thus, highlighting the complex interplay between dedication, exhaustion, and the emotional burden of caregiving.

*“I am also growing so there are times I don’t feel well. Even yesterday; the day before yesterday I was not feeling fine but I have to get up and take care of her. I have to forget my own sickness and fatigue and everything to be able to take care of her”* (Daughter of PwD attending Manhyia government hospital, 45–49 years).

*“It takes a lot of my time, as you know a lot of patients attend Tafo hospital, we sometimes get to the hospital at 5am. I feel burdened when I am supposed to take her to the hospital the following day. It worries me a lot but there is nothing I can do about it”* (Daughter of PwD attending Tafo government hospital, 45–49 years).

Another participant narrated:

*“I go through a lot of challenges. For instance, because I have to go with her to work every day, I get very fatigued multitasking. Sometimes, I get home in the evening feeling very tired… I get discouraged sometimes, I sometimes feel like giving up, I sometimes feel burdened a lot”* (Daughter of PwD attending Tafo government hospital, 30–34 years).

### Experience of physical and verbal abuse, and stigma

A few participants recounted moments of physical and verbal abuse as a result of the intermittent aggressiveness of the PwD. There were reports of some caregivers having their clothes torn, others being insulted and cursed by the PwD. Even though some people understood that the attitude was a result of the disease, the abuse still had an impact on them. The emotional toll of the abuse was evident as caregivers expressed feelings of sadness, frustration, and helplessness. Many struggled with conflicting emotions, as they grappled with their commitment to providing care while also protecting their own wellbeing.

*“Yes, sometime past, he held me and my kids said they would call police men to arrest him, but he wouldn’t listen, he still wanted to hurt me.”* (Daughter of PwD attending University Hospital– KNUST, 50–54 years).

Another participant expressed similar sentiments:

*“Aww sometimes, he claims that I am a witch, and he insults me. I can even try calming him down, but to no avail and sometimes I even cry. Some people may understand that it is as a result of his sickness, but others may also not take it lightly when they hear him claiming that I am a witch. It is a whole lot of issues, and sometimes I become worried.”* (Daughter of PwD attending University Hospital– KNUST, 45–49 years).

Some participants suffer from secondary stigma or stigma by association as they cared for their family members with dementia. A few respondents reported shame brought upon them by the symptoms of PwD which potentially made them lose respect in the community. According to a participant;

*“It is sometimes shameful because the respect which the neighbours had for us in society diminishes because they used to hold us in high esteem and now, we have a mother that is like this; it can be very shameful. Sometimes I feel ashamed when she acts abnormally. I do not want anyone to know that this is what happens.”* (Daughter of PwD attending Ejisu government hospital, 25–34 years).

*“They behave abnormally. She would say someone wants to kill her and even accuse me of conniving with the person to come and kill her. Sometimes she will pour her urine from her chamber pot in the morning in front of the house and say that I have hidden some black magic charms there, and so, her urine can destroy it. She was very aggressive; she even fought with our family friend hitting her head against the wall and accusing her of conniving with me to kill her. So, sometimes, I feel ashamed when she behaves like that”* (Son of PwD attending Kumasi South Hospital, 45–49 years).

### Perceived blessings from caregiving

Surprisingly, our study revealed that being a caregiver for a PwD was not exclusively associated with negative impacts. The participants expressed that since taking on the caregiving role, they had not experienced any illness. They attributed this physical well-being to the blessings that they believed accompany the act of caring for those in need. This view emphasized a deep sense of spiritual purpose and protection that resonated with their caregiving journey. Furthermore, the caregiver extended this belief to their work environment, perceiving a divine presence that accompanied them not only in caregiving but also in their daily work activities. For the participants, caring for PwD could be described as a blessing. The following quotes reflect this finding:

*“Since I started caring for her, by God’s grace, I have never been ill. I think that it is the blessings that comes with caring for sick people. And God is with me at work too”* (Son of PwD attending Manhyia government hospital, 30–34 years).

*“I have been blessed by God, I attend Deeper Life church, I think I am always fit to do everything. I do not fall sick at all, even my children do not get sick. God has blessed me”* (Wife of PwD attending Onwe government hospital, 50–54 years).

There was another participant who contended that:

*“Since I care about her wholeheartedly my life has improved, even though the work I do is nothing big, I am able to provide for my children because I care for her. I would say that by the grace of God, my work is improving and I am able to provide for my children. So those are the changes I have observed”* (Daughter of PwD attending Tafo government hospital, 30–34 years).

## Discussion

This study sought to explore the perspectives of primary caregivers about the impact of caring for PwD in Ghana. The findings from our study provide valuable insights into the current understanding of caregiving for PwD in Ghana.Our findings that caregivers had to sacrifice their personal interests and struggle with making time commitments aligns with previous studies [6, 26] that have found impaired social relationships and engagement among caregivers of PwD. For instance, our study is corroborated by Brodaty and Donkin [6] who argue that caregivers often make sacrifices in terms of their leisure activities and interests, limit their time spent with friends and families. Similar findings have been reported in Singapore [27]. A possible explanation for this finding could be that the decision to sacrifice personal interests may stem from an altruistic desire to monitor and ensure the safety and wellbeing of the PwD. Another perspective is that PwD often experience difficulties in communication, making it challenging for caregivers to understand their needs and preferences. This can require increased efforts and time, leaving the caregivers with little opportunity for personal leisure. Hence, the sacrifice of personal interests.

Caring for PwD was found to financially strain the caregiver while impacting negatively on their jobs. Our finding is corroborated by previous studies conducted in South Africa [20], Uganda [28], and China [29]. The observed financial strains often arise as a result of the high cost of medications for the PwD as well as auxiliary health expenditure such as dietary needs of the care recipient and the cost of transportation to the health facilities [20]. These financial demands can quickly accumulate, leading to strain on caregivers’ budgets. Consequently, caregivers allocate a significant portion of their income to cover these costs, which disrupts their financial stability and impact their ability to meet other financial obligations. This strain can also result from reduced work hours or even the need to leave employment altogether to provide full-time care as observed in the narrations of the caregivers.

Our study revealed that caregivers of PwD were consistently overwhelmed by stress and experienced burnout– a result that is consistent with a study conducted in Shanghai, China [17]. Other studies conducted in Hong Kong [30] and Canada [31] support our findings. Mainly, this stress and fatigue was attributed to the role of multi-tasking and putting the needs of the PwD ahead of the caregiver. The repetitive nature of caregiving tasks, coupled with a lack of time for self-care may also explain the overwhelming stress and burnout that was reported by the caregivers. The foregoing evidence highlights a need for primary caregivers to prioritize self-care practices as they navigate through the strains of caregiving. Furthermore, the study underscores a need for healthcare providers to encourage primary caregivers of PwD to intermittently take breaks to recharge, as well as provide them with practical tips on incorporating self-care routines into their daily lives.

Another notable finding from our study was the profound emotional and psychological challenges faced by caregivers of PwD, including the experience of physical and verbal abuse and the burden of secondary stigma. This finding is congruent with earlier studies reported in the United States of America [34 and South Africa [33]. Caregivers described how the symptoms of PwD, particularly their behavioural changes, could bring shame and lead to a loss of respect within their communities. Many caregivers found it challenging to manage their own feelings of embarrassment and shame when their loved ones exhibited abnormal behaviours associated with dementia. This experience can further isolate caregivers and complicate the caregiving journey. Hence, building more compassionate communities would help to reduce abuse and secondary stigmatisation of primary caregivers of PwD.

Amidst the negative impacts contended by caregivers, we found an unexpected finding that caregiving was perceived to be associated with blessings. Primarily, the blessing was perceived to be manifested in good health and non-hospitalization of the caregiver. The perception of caregiving as a source of blessings suggests that caregivers find positive meaning and fulfilment in their role, despite the hardships. Our finding is supported by an earlier study that has reported that caregivers in Ghana believe that their role brings about blessings [34]. This positive outlook might arise from cultural or spiritual beliefs, personal values, or a deep sense of purpose. Traditionally, Ghanaians are socialised to believe that it is responsibility to care for their sick relatives just as they did for them in their childhood. It is believed that by fulfilling this obligation to the sick, including family member living with dementia, the caregiver would attract the blessings of God and the ancestors [34].

### Policy implications

Our findings regarding the financial strains of caregiving underscores a need for the government of Ghana through its agency (i.e., the National Health Insurance Authority) expand the scope of the national health insurance scheme to cover the full cost of dementia care. Additionally, the national health insurance policy must be amended to make room for it to cover psychological health needs of primary caregivers of PwD. Our findings provide evidence for the implementation of comprehensive support mechanisms to address caregivers’ emotional well-being. Practically, interventions such as counselling, support groups, and respite care must be implemented in all healthcare facilities to ensure the caregivers receive the necessary psychological support to ease their stress. This study underscores a need to build more compassionate communities in the rural settings of Ghana. Compassionate communities can offer resources, education, and support to caregivers, which can reduce the likelihood of abuse and neglect of PwD by helping caregivers cope with their own emotional and psychological stress. Currently, there are already compassionate communities such as Alzheimer’s Ghana, a non-governmental organisation dedicated to combating dementia stigma and providing essential training programs for caregivers. Through public awareness campaigns and caregiver training, Alzheimer’s Ghana has made significant strides in supporting caregivers and raising awareness about dementia-related issues [35]. To further expand the reach of such initiatives, it is crucial for the government to provide substantial financial resources and logistical support. By investing in organisations like Alzheimer’s Ghana and enabling them to extend their services to rural communities, where caregivers often face significant psychological stress, Ghana can work towards building more compassionate communities and providing essential support to those caring for PwD.

### Strengths and limitations

Adopting a descriptive phenomenological design was a strength for our study as it helped us to capture the lived experiences of the primary caregivers of PwD. The study not only examines the negative impacts of caregiving but also uncovers an unexpected finding related to caregivers perceiving caregiving as associated with blessings. This comprehensive approach adds depth to the analysis and contributes to a nuanced understanding of the emotional complexities of caregiving. Notwithstanding, there were some limitations that must be considered. First, the study was limited to only unpaid primary caregivers of PwD who visited the healthcare facilities where the study was conducted. Hence, the findings might not reflect caregivers whose care recipients did not receive care from any of the healthcare facilities included in the study, as well as paid caregivers. Also, there is a possibility of self-reported bias since participants were recounting their lived experiences.

## Conclusion

The study’s findings resonate with existing literature, highlighting the consistent struggles faced by caregivers. Sacrificing personal interests, navigating financial strains, and grappling with stress and burnout emerged as pervasive themes. We conclude that despite the negative impacts of caring for PwD, caregivers perceived their role as associated with blessings, deriving positive meaning and fulfilment from their caregiving journey. This study underscores a need to build more compassionate communities in the rural settings of Ghana.

### Electronic supplementary material

Below is the link to the electronic supplementary material.

Supplementary Material 1

## Data Availability

The data used for the analysis is freely available from the first author upon reasonable request.

## Abbreviations

GHS-ERC: Ghana Health Service Ethics Review Committee
KNUST: Kwame Nkrumah University of Science and Technology
LMICs: Low-and-middle-income Countries
PwD: Persons Living with Dementia
NHIA: National Health Insurance Authority
SSA: Sub-Saharan Africa
WHO: World Health Organization
