# Supplementary material for: Primary caregivers’ experiences of caring for people living with dementia in Ghana: a phenomenological study
Source: BMC Geriatr. 2024 Apr 1;24:304. doi: 10.1186/s12877-024-04894-6 (PMC10985993; doi:10.1186/s12877-024-04894-6)
Supplement: Supplementary file 1 — Supplementary Material 1 [file 12877_2024_4894_MOESM1_ESM.docx]

# **PREVALENCE, PREDICTORS AND EXPERIENCES OF PEOPLE LIVING WITH DEMENTIA IN GHANA**

# **SEMI-STRUCTURED INTERVIEW GUIDE**

**Introduction**

Hello (name of participant), I’d like to welcome you and say thank you for your time today. I recognize that we both have busy schedules and we are hoping to get as much as possible from this interview. I anticipate that this interview will last about half an hour.

The purpose of this interview is to gain deeper understanding of your experiences as a family caregiver of a person living with dementia. Today, I am interested in hearing your experiences, the changes that have occurred in your life as a result of this condition, the challenges you encounter, and the coping strategies that you adopt.

This interview will be recorded and then transcribed for analysis. There will be no names attached to the transcripts so your words will remain confidential. I have a series of questions that I would like to ask to prompt your responses, examples and stories about your caregiving experience with dementia; there are no right or wrong answers, just your own thoughts and insights. Again, thank you so much for your participation. If you are ready, I will begin with the first question. [***turn on audio recorder***]

**Section A: Background characteristics of participants**

| **1. Gender:**  🞏 Male                         🞏 Female          🞏Other (specify): _______ | | **2. Age (in years):** _____  🞏 15-24                🞏 25-34            🞏 35-44          🞏 ≥45 |
| --- | --- | --- |
| **3. Ethnicity:**  🞏 Akan  🞏 Ewe  🞏 Ga-Dangme  🞏Other (specify):  ______ | **4.Marital status:**  🞏Single  🞏Married  🞏 Divorced  🞏 Separated  🞏 Other (specify): ________________ | **5. Educational level:** How much schooling have you had?          🞏 No formal education          🞏 Graduated from primary school          🞏 Graduated from junior high school (JSS/JHS)          🞏 Graduated from senior high school (SSS/SHS)          🞏 Graduated from tertiary institution |
| **6. Religion:** 🞏 Christianity 🞏 Islam 🞏 Traditional 🞏 Other (please specify): ______________ | | |
| **7. Rural-urban residence:**   🞏 Rural                  🞏 Urban  🞏 Semi-urban | | |
| **8. a. Employment**: Do you work?  🞏 Yes  🞏 No  b. If yes, you work as:    🞏 full-time     🞏 part-time 🞏 Other: __________ | | |
| **9. Household wealth index**  9a. What is the main source of drinking water for members of your  household? **(Tick only one)**  🞏 Piped-inside house/yard 🞏 Piped-public tap/kiosk  🞏 Borehole/Well 🞏 Rainwater  🞏 Neighbour’s tap         🞏 Flowing river/stream  🞏 Dam/Stagnant water   🞏 Other (specify): _________ | | 9b. What type of toilet facilities do members of your household mainly use? **(Tick only one)**  🞏 Flush toilet             🞏 VIP  🞏 Latrine   🞏 Bucket/chemical toilet  🞏 Flowing river/stream     🞏 No facility (bush)  🞏 Neighbour’s toilet    🞏 Other (specify): _________ |
| 9c. What type of fuel does your household mainly use for cooking?  **(Tick all that apply)**  🞏 Electricity from generator 🞏 Gas (LPG)  🞏 Wood       🞏 Electricity from grid  🞏 Coal / charcoal 🞏 Kerosene/paraffin  🞏 Electricity from solar energy 🞏 Other (specify): _________ | | 9d. Does anyone in your household have any of the following in good working condition? **(Tick all that apply)**  🞏 Bicycle 🞏 Gas cooker 🞏 Radio  🞏 Fridge/freezer  🞏 Mobile/cellular telephone 🞏 TV  🞏 Sofa/sofa set 🞏 Video recorder/DVD player  🞏 Other (specify): __________ |
| 9e. Does your household have any of the following domestic  animals/fowl? **(Tick all that apply)**  🞏 Cows   🞏 Goats/sheep 🞏 Pigs  🞏 Chickens/ducks   🞏 Rabbits  🞏 Other (specify): _______ | | 9f. Please tell me, what is the main source of your household’s income? By that I mean, from which source does most of the money used in this household come from? **(Tick only one)**  🞏 Self-employed, informal jobs, selling or trading  🞏 Wages/salary from formal job  🞏 Government grants  🞏 Income from rental property  🞏 Retirement fund  🞏 No source of income  🞏 Other (specify): ________________________ |
| 9g. Compared to 3 years ago, would you say your financial situation is better or worse?  🞏 Better     🞏 About the same   🞏 Worse off | | |
| **10. How many people do you live with in your household?**  🞏 I live alone       🞏 1           🞏 2           🞏 3           🞏 4       🞏 ≥5 | | **11. Are you on health insurance?**  🞏 Insured              🞏 Uninsured |

**Section B: Experiences of with dementia**

1. Could you please tell me about your experience of dementia?

**Probe**: understanding about the causes of dementia, stigma, care arrangement, health-seeking behaviours.

1. What are your experiences with the spiritual care of your relative who is living with dementia at home (or in the nursing home)?

**Probe**: a. Can you recall more details of a specific incident?

b. Do you have further examples of this?

1. What changes have occurred in your life because of caring for your relative with dementia?

**Probe**: a. Social, economic, and health-related changes.

b. Can you recall more details of a specific incident?

c. Do you have further examples of this?

**Section C: Challenges and coping strategies**

1. What are the challenges you face in caring for, interacting with, and handling the changed behaviours of your relative with dementia at home?
2. Please describe an example where it was particularly challenging for you to care for your loved one.

**Probe**: Please explain further why that situation was so challenging compared to other days/situations?

1. What strategies do you use in coping with the challenges of caring for your relative and dealing with your relative’s changed behaviours?
2. Please give a detailed example of a time when a strategy worked well for managing a challenging behaviour or rendered care.

**Probe**: Please provide your insights about why you think this strategy worked.

1. Please describe an example of when a particular strategy did not work well?

**Probe**: Please provide your insights about why you think this strategy did not work well.

**Section D: Closing courtesies**

1. Are there any corrections, additions or other comments that you would like to share or what you believe to be the key takeaways from this interview?

Thank you for participating today. I truly appreciate your time and unique insights; your contributions today have been truly valuable. Is there anything else that you would like to share?

If not, again thank you for joining today and sharing your insights with me [**recording will be turned off**].
